# Aging in iPS cells

**DOI:** 10.18632/aging.100653

**Published:** 2014-04-27

**Authors:** Justine Miller, Lorenz Studer

**Affiliations:** Sloan-Kettering Institute for Cancer Research

Human induced pluripotent stem cell (iPSC) technology [1] holds the promise of recreating disease *in vitro* in a patient-specific manner. There has been considerable success in using iPSC models to study early-onset genetic diseases, such as our own work on familial dysautonomia [2] or primary herpes simplex encephalitis [3]. The modeling of late-onset diseases in iPSC-derived lineages has been more challenging and such studies often report phenotypes that inadequately recapitulate the disease (reviewed in [4]). Our recent study [5] demonstrates the failure of patient-specific donor cells to maintain age-associated markers during iPSC reprogramming and subsequent differentiation. Such rejuvenated iPSC-derived lineages therefore may not be suitable to model late-onset disease. Taking cues from a premature aging disorder known as Hutchinson-Gilford progeria syndrome (HGPS), we present a strategy for reintroducing age-like features in iPSC-derivatives towards the more faithful modeling of late-onset disease.

Age is the most important risk factor in many late-onset disorders such as Parkinson's disease (PD) as illustrated by the fact that PD patients do not develop symptoms until later in life. Therefore, it is imperative to consider age as well as genetic mutations when attempting to model these diseases *in vitro*. Previously, it was unclear whether a donor cell from an old individual would maintain its age-associated properties following conversion into other cell fates *ex vivo*. However, recent studies have presented evidence that markers of cellular age, including mitochondrial fitness and telomere length, are reset to a young-like state when old donor fibroblasts are reprogrammed to iPSCs (reviewed in [6]). Indeed, our own study defines a broad set of age-associated markers, and we demonstrate the rejuvenation of old donor fibroblasts based on those markers. The corresponding iPSCs derived from old donors no longer exhibit features that distinguish old from young primary cells including abnormal nuclear morphologies, accumulated DNA damage, increased reactive oxygen specifies (ROS), reduced levels of a set of nuclear organization proteins, and loss of heterochromatin markers. We could not be sure, however, whether pluripotency simply suppresses “age” by downregulating age-related proteins such as progerin. Indeed HGPS iPSCs also show a loss of the age-associated markers at the pluripotency stage. Therefore, iPSCs were differentiated into a fibroblast-like cell in order to match the phenotype of the donor fibroblasts used for reprogramming. We were able to show that similar to the pluripotency stage, iPSC-derived fibroblasts from old donors appear “young”, suggesting that the cell's intrinsic molecular clock is reset following the reprogramming step. In contrast, HGPS iPSC-derived fibroblasts quickly upregulate progerin (the disease-causing protein) during differentiation, resulting in the re-induction of age-associated phenotypes. Based on these findings we hypothesized then that the difficulties of modeling late-onset disease in differentiated iPSCs could be caused by the fact that they are too “young” and that the implementation of defined genetic cues such as progerin overexpression may be sufficient to reintroduce age-associated markers.

Using synthetic mRNA technology [7] we observed that progerin overexpression returns old donor iPSC-derived fibroblasts to an aged-like state that resembles the profile of the original fibroblasts. Furthermore, progerin overexpression in iPSC-derived midbrain dopamine (mDA) neurons, the cell predominantly affected in PD, not only induces abnormal nuclear morphologies and accumulation of DNA damage and ROS, but it also drives processes more specific to neuronal aging. Importantly, progerin-aged mDA neurons have shorter dendrites, and progression of the phenotype follows the classical dying-back model observed in the aging brain [8] distinct from cellular pathologies following an acute toxic insult. Furthermore, progerin elicits gene expression changes compatible with a neuro-degenerative process and drives the accumulation of neuromelanin, an mDA neuron-specific, age-related pigment.

We then wondered whether introducing an age-like component in iPSC-derived mDA neurons from PD patients would synergize with the genetic vulnerability of these patients to yield relevant late-onset phenotypes. Indeed, short-term progerin overexpression induces enhanced dendrite shortening, increased cell death, and AKT dysregulation in a PD-specific manner. Upon extended exposure, progerin also triggers the loss of tyrosine hydroxylase and induces the formation of inclusion bodies, mimicking disease progression.

Our study represents the first attempt at programming cellular age in iPSC-derived lineages, and as such, many important questions remain unanswered. Is age truly re-set or could reprogramming select for a young-like cell among the old donor cells? Is progerin-induced aging in fibroblasts of neurons reversible? Does the more nuanced manipulation of progerin levels induce cells to adopt an intermediate age range more defined than “old” versus “young”? Could exposure to low levels of progerin affect cell maturation? Can progerin-induced aging be applied to any late-onset disease model or is it restricted to certain lineages and disease conditions? How closely does progerin mimic the normal aging process and are there alternative strategies that may better phenocopy the aging process? The answers to these questions will be critical for modeling both age and disease in a dish. The work could lead to a future where it is possible to test-run an individual's susceptibility to age-dependent diseases across many iPSC-derived lineages. Such technologies may ultimately allow us to preempt disease or to develop individualized therapies even prior to disease onset, heralding a new category of personal medicine unimaginable just a few years ago.
